# Nanoscale hybrid systems based on carbon nanotubes for biological sensing and control

**DOI:** 10.1042/BSR20160330

**Published:** 2017-03-02

**Authors:** Youngtak Cho, Narae Shin, Daesan Kim, Jae Yeol Park, Seunghun Hong

**Affiliations:** 1 Department of Physics and Astronomy, Seoul National University, Seoul 151-747, Korea; 2 Department of Biophysics and Chemical Biology, Seoul National University, Seoul 151-747, Korea; 3 Department of Automotive Engineering, Doowon Technical University College, Anseong 456-718, Korea

**Keywords:** biointerface, carbon nanotube, cell controll, functionalization, surface-programmed assembly, sensing

## Abstract

This paper provides a concise review on the recent development of nanoscale hybrid systems based on carbon nanotubes (CNTs) for biological sensing and control. CNT-based hybrid systems have been intensively studied for versatile applications of biological interfaces such as sensing, cell therapy and tissue regeneration. Recent advances in nanobiotechnology not only enable the fabrication of highly sensitive biosensors at nanoscale but also allow the applications in the controls of cell growth and differentiation. This review describes the fabrication methods of such CNT-based hybrid systems and their applications in biosensing and cell controls.

## Introduction

A carbon nanotube (CNT) is a rolled-up graphene that is carbon atoms arranged in a planar honeycomb lattice [1]. CNTs can be classified into single-walled CNT (swCNT) and multi-walled CNT (mwCNT) depending on the number of rolled-up graphene layers. Generally, the diameter of swCNT is approximately 1–2 nm and that of mwCNT varies from 2 nm to over 100 nm. Due to electrically, mechanically and chemically unique properties of CNTs [2–4], CNTs have been widely utilized in electronics, optics, materials sciences and biology [5,6].

During the last decades, CNT-based nanoscale hybrid systems have been intensively studied for biological interface applications [7–9]. For example recent advances of versatile nanofabrication methods such as surface-programmed assembly enable the fabrication of highly sensitive and selective biosensors at nanoscale [7,10,11]. CNT-based biosensors functionalized with biomolecules, such as enzyme, antibody and olfactory receptor, can detect target molecules at a femto-molar concentration with the benefit of biocompatibility [12–14]. As an example, an olfactory receptor-immobilized biosensor can selectively detect a target odorant at <10 fM concentration without responding to non-targeted odorants [12]. Furthermore, a CNT-based biosensor fabricated at the end of a nanoneedle can monitor the intracellular calcium concentration of living cells, which allows us to better understand the calcium signal pathways [15]. CNT-based biological sensors also have been applied for the detection of versatile biological substances such as antibiotics, environmental hormones, drugs and odorants [10,16–18].

On the other hand, since CNTs are composed of carbon atoms and they are chemically inert, CNT-based devices do not release any toxic ions at the interface between the CNTs and biological tissues [19]. This chemically stable property of CNT provides great advantages in the applications of cell control and tissue regeneration using CNT-based hybrid devices. Furthermore, surface-programmed assembly techniques enable the mass production of aligned- or patterned-CNT networks on any dimensional structures for the control of cell growth and differentiation [11,20–22], indicating the applications of CNT-based hybrid systems for improved clinical performances in tissue regeneration [9,23–25]. For instance CNT-based line-shaped patterns with its width of 20 μm were found to enhance the differentiation of human mesenchymal stem cells (hMSCs) to neural lineages [26].

In this review, we will discuss the *fabrication methods of CNT-based nanoscale hybrid systems* for biological interface, especially focusing on the engineering methods for combining CNTs with biological systems. Furthermore, we will discuss possible applications of CNT-based hybrid systems for *biomolecular detections* and *cell controls*.

## Patterning and functionalization of CNT-based hybrid systems on solid substrates

In general, CNTs can be synthesized by several methods including arc discharge, laser ablation, high-pressure carbon monoxide (HiPco) and chemical vapour deposition (CVD) [27–32]. In this chapter, we will discuss the methods to pattern and functionalize CNT-based hybrid systems on solid substrates to build versatile functional devices.

### Preparation of CNT-based hybrid systems on solid substrates

**Figure 1:**
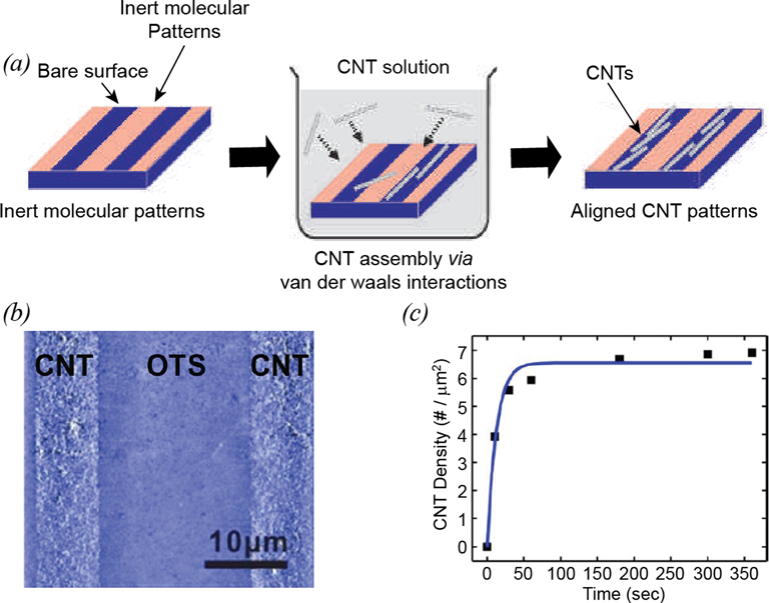
Surface-programmed assembly (**a**) Schematic diagram of CNT assembly on to a molecular patterned substrate [14]. (**b**) AFM images of CNT-octadecyltrichlorosilane (OTS) patterned substrates [33 ]. (**c**) Graph showing the number density of CNTs adsorbed on to 3 μm × 3 μm and the theoretical fitting (solid line) based on the Langmuir isotherm [20].

Many researchers have developed various methods for the patterning of CNTs on solid substrates. One of the useful methods for CNT patterning is *surface-programmed assembly* method, which utilizes internal molecular forces to direct the assembly processes of CNTs on solid substrates. The surface-programmed assembly method generally includes two major steps; molecular patterning and CNT self-assembly. In the molecular patterning process, substrates are first patterned with self-assembled monolayer (SAM) molecules to provide polar and non-polar regions. Here, micro-contact printing, dip-pen nanolithography and photolithography are utilized for SAM patterning. For the selective assembly of CNTs, the SAM-patterned substrate is placed into a CNT solution. Due to the van der Waals interactions between the CNTs and the polar region, the CNTs are selectively adsorbed on to the polar regions of the substrate, while the non-polar surface blocks the CNT adsorption (Figure 1a,b) [20]. In this case, the assembled structures of CNTs can be completely determined by the shape of the SAM patterns. One interesting phenomenon related to this CNT assembly processes is “self-limiting mechanism”. In a CNT suspension, CNTs are first adsorbed on to the polar surface very quickly. However, the number of adsorbed CNTs is saturated over time because the pre-adsorbed CNTs completely block the additional adsorption of CNTs (Figure 1c). This self-limiting mechanism allows one to prepare a uniform layer of CNTs throughout the substrate. Since the CNTs are stably adsorbed on the surface, additional microfabrication processes can be performed to fabricate various functional devices such as field-effect transistors (FETs), cell growth patterns and sensor transducers. Moreover, the surface-programmed assembly method can be very useful for practical bio-applications because the entire processes can be carried out at room temperature using conventional microfabrication facilities.

External guide forces, such as fluid flow and electric field, have been utilized to improve the precision of CNT assembly on solid substrates. Huang et al. [34] reported the assembly method using liquid flows to control the alignment of nanowires. The authors assembled the arrays of CNTs by flowing a CNT suspension through a fluidic channel of polydimethylsiloxane (PDMS) in contact with a molecular patterned substrate. Dielectrophoresis-based method can be utilized to control the assembly of CNTs between two electrodes. Chen et al. [35] aligned CNTs using an electric field induced by applying an alternating current (AC) between the two electrodes. In a CNT suspension, CNTs are attracted towards the high electric field regions between two electrodes via dielectrophoretic forces and they assembled across the electrodes. Fan et al. [36] utilized catalyst patterns to selectively grow CNTs at specific locations on substrates. From micrometre-sized catalyst islands, CNTs were grown by CVD. Furthermore, the growth direction of CNTs can be controlled by applying gas flow during the growth [33 ]. These works allow the synthesis and understanding of well-aligned nanotubes with uniform diameters on the substrate.

### Functionalization of CNTs for biological interfaces

The biomolecule-immobilized CNTs can be utilized to build highly selective biosensors and tissue regeneration scaffolds. However, since the surfaces of CNTs are rather chemically inert, it is often very difficult to fix functional biomolecules on CNT surfaces. In this section, CNT functionalization methods including oxidation and linker-mediated modification processes will be discussed.

**Figure 2:**
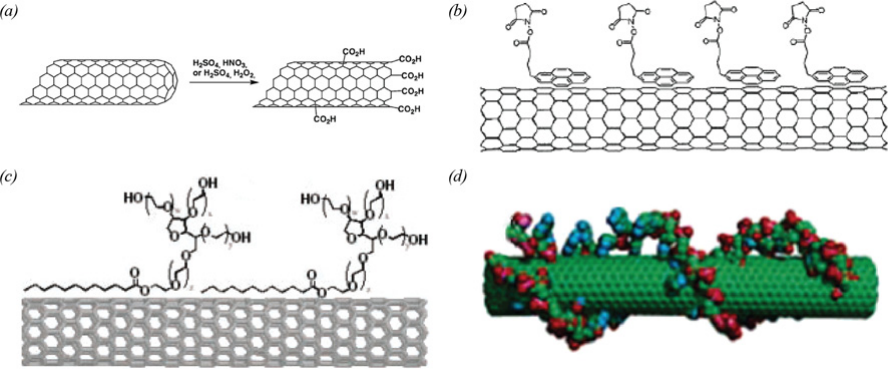
Functionalization of CNTs (**a**) Strong acid treatments and their effects on CNT surfaces [40 ]. (**b**) Chemical linker-based functionalization with pyrene moieties [35 ]. (**c**) Surfactant (Tween 20) modification on CNT surfaces [41 ]. (**d**) DNA-direct assembly of CNTs [42].

Oxidation of CNT surfaces creates defects at the ends and on the side walls of CNTs. Then, chemical functional groups can be induced on the defects so that biomolecules are linked to the functional groups. One method for the oxidation of CNT surfaces is a strong acid treatment. In this method, CNTs were immersed into the mixed solution of nitric and sulfuric acids. Then, the acid mixture is heated at approximately 110°C. Finally, the CNTs were rigorously washed with deionized water and then dried. During the oxidation processes, the defects are introduced on to the surface of CNTs. Then, oxygen containing functional groups, such as carboxylic acids and aldehydes, are introduced on to the defect sites (Figure 2a). Especially, carboxylic acids can be easily cross-linked with amine groups via amide bonds. Feigel et al. [37] reported that DNAs containing primary amines can be immobilized on to CNTs, which have carboxyl groups after oxidation process. The immobilization of antibodies with abundant amine groups on the oxidized CNTs was studied by Yu et al. [38]. Another oxidation method of CNTs is oxygen plasma treatment. In this method, CNTs are exposed to oxygen plasma. Here, the plasma produces defects in CNTs and also dissociates oxygen molecules. The dissociated oxygen molecules react with the defects, functionalizing CNTs with oxygen-containing chemical groups. Fernandez et al. [39] utilize the oxygen plasma method for ferritin immobilization. However, oxidation processes can alter the electrical properties of CNTs due to the generation of numerous defects on CNT surfaces.

Chemical linker molecules can be coated on CNT surfaces to introduce high-density surface functional groups for biomolecular binding, while preserving physical structures of CNTs. For example a functionalization method for CNTs using 1-pyrenebutanoic acid succinimidyl ester (PSE) was reported by Chen et al. [43]. PSE is a bifunctional molecule that consists of pyrenyl terminal group and succinimidyl moiety as another terminal part. When CNTs were placed in PSE solution, the pyrenyl groups of the PSEs adhered on the side walls of CNTs via aromatic-aromatic interaction (π-stacking) (Figure 2b). Once the PSEs bind on to the CNT surfaces, the succinimidyl moieties of the PSEs can be substituted with amine groups from protein bindings. Based on this process, the authors immobilized ferritins and streptavidins on to the side wall of CNTs. Using a similar strategy, glucose oxidase [13] and haemoglobin molecules [44] were immobilized on the surface of CNTs.

Various surfactants have been utilized for CNT functionalization. A CNT functionalization process using a modified surfactant, biotinylated Tween, was reported by Chen et al. [41]. A biotinylated Tween molecule consists of a linear aliphatic chain and three biotinylated moieties. When CNTs were suspended in a biotinylated Tween aqueous solution, the linear aliphatic chain of biotinylated Tween non-covalently adhered to the side walls of CNTs via van der Waals interaction (Figure 2c). Using this method, the authors investigated the binding of streptavidins on to biotinylated Tween-modified CNTs. Shim et al. [45] performed a CNT functionalization strategy using other surfactant, Triton-X100, as a linker for the immobilization of streptavidin.

On the other hands, CNTs can also be functionalized by the direct incorporation of biomolecules without oxidation or linker-mediated modification processes. The direct functionalization of CNTs with DNA was studied by Lu et al. [42] (Figure 2d). In this study, CNTs were placed in poly-T DNA solution, and the solution was gently sonicated for DNA molecules to wrap CNTs via π-stacking. In addition, Guo et al. [46] immobilized the protein metallothionein on the surface of CNTs. Since the protein containing histidine and tryptophan exhibits specific affinity on to the CNTs, the protein can be directly attached on the CNT surfaces.

## Sensing

CNTs have great advantages in building highly sensitive sensors due to their unique structures and electrical properties. In addition, the mechanical stabilities and chemically inert properties of CNTs are also advantageous for the applications of ultrasensitive biosensors. In this chapter, the biosensor applications of CNT-based nanoscale hybrid structures will be discussed.

### Mechanism of CNT transistor-based sensors

**Figure 3:**
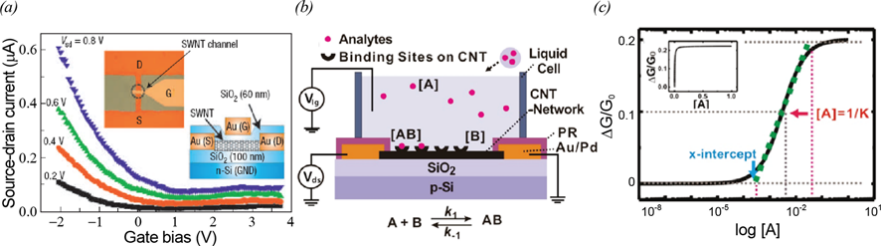
Basic mechanism of chemical and biological sensors based on CNT-FETs (**a**) Gating effect of a top-gate CNT-FET under different source-drain voltages. The insets show the optical image (left) and cross-sectional structure (right) of a fabricated CNT device where S, D and G are the source, drain and gate electrode respectively [11]. (**b**) Schematic diagram depicting the experimental set-up using a CNT-FET. The [A], [B] and [AB] represent the concentration of analytes in bulk solution, the surface density of binding sites and the surface density of adsorbed analytes respectively [47 ]. (**c**) Schematic diagram depicting the theoretical modelling for CNT-based sensors. It is assumed that analytes A are adsorbed to the binding sites B following the Langmuir isotherm process. Then, adsorbed analytes AB generate the sensor response, ΔG/G_0_ [47].

The detailed structure of a CNT-FET and its gating effect are shown in Figure 3a. CNT-FETs exhibit typical p-type behaviours, where source-drain currents decrease with an increasing gate bias.

Figure 3b shows schematic diagrams depicting the mechanism and the theoretical model of the CNT-based sensors [48]. CNT-based transducer devices were coated with specific receptor molecules with binding sites (B) which can selectively bind to target molecules (A). Typically, the binding events between analytes A in the bulk solution and the binding sites B on the CNT surfaces follow the Langmuir isotherm model. In the Langmuir isotherm model, it is assumed that analytes bind to a finite number of binding sites on sensor surfaces, and the binding analytes and non-binding analytes maintain an equilibrium state in general. Here, one can choose different binding sites depending on target analytes. For example the binding sites of CNT-based gas sensors can be bare surfaces of CNTs in the device channels [49–51]. In the case of CNT-based biosensors, specific receptor molecules fixed on CNT surfaces provide binding sites [13]. In Figure 3b,c, *[A]*, *[B]*, *[AB]* and *[B]*_max_ represent *the concentration of analytes in the bulk solution*, *the surface density of the binding sites on CNTs*, *the surface density of adsorbed analyte molecules* and *the maximum surface density of the binding sites on CNTs* respectively. In this case, the surface density of adsorbed analytes can be expressed by the Langmuir isotherm equation like

$$\;\left[\mathrm{AB}\right]={\left[B\right]}_{\max}\;\times \frac{\left[A\right]}{\left[A\right]+1/K}$$

with the equilibrium constant *K = k*_1_/*k*_-1_.Here, *k*_1_ and *k*_-1_ are the *association* and *dissociation* constants respectively. If we assume that the conductance changes of a CNT transistor transducer (*ΔG*) are linearly proportional to the number of adsorbed analytes, the sensor sensitivity │*ΔG/G_0_* │ of the CNT transistor transducer can be approximated as │*ΔG/G_0_* │ = *k*[AB], where k is a constant representing the response characteristics of the CNT transistor transducer. Thus, we can write the sensitivity │*ΔG/G_0_* │ of the CNT transistor transducer like

$$\;\left|\mathrm{\Delta G}/G_{0}\right|=\;k{\left[B\right]}_{\max}\times \frac{\left[A\right]}{\left[A\right]+1/K}$$

Thus, by fitting measured data using the equation, we can estimate the equilibrium constant *K* between [A] and [B].

### CNT-based sensors

#### Sensors based on bare CNT

**Figure 4:**
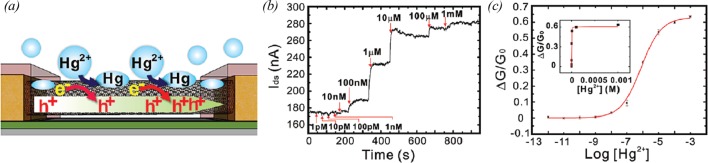
Ion sensors comprised CNT-based hybrid nanostructures (**a**) Plausible mechanisms for Hg^2+^ detection [52]. (**b**) Real-time current measurement obtained from a CNT-FET sensor after the injection of Hg^2+^ solutions at various concentrations [52]. (**c**) Conductance change of the CNT-FET sensor by the injection of Hg^2+^ solutions at various concentrations. The red line indicates the fitting curve for the estimation of equilibrium constant [52].

The extremely large surface areas of CNTs are favourable for the adsorption of molecules. In addition, CNTs exhibit significant changes in their conductance when small molecules are adsorbed. Based on these properties, CNT-FETs without biomolecular coatings have been employed to detect specific molecules. For example Kim et al. [52] developed Hg^2+^ sensors based on the redox reaction between CNTs and Hg^2+^ ions (Figure 4a). They employed the mechanism that the adsorption of Hg^2+^ ions on the surface of CNTs lead to the reduction in the Hg^2+^ ions and the oxidation of the CNTs. In this process, the CNTs give electrons to the Hg^2+^ ions, and then, consequential hole injections cause conductance increase in CNT junctions due to the *p-type* characteristics of CNTs. Using the Hg^2+^ sensors, the authors can detect the injection of 10 nM Hg^2+^ solution that corresponds to the maximum allowable level of Hg^2+^ ions in drinking water according to an environmental protection agency regulation (Figure 4b). The calibration curves show that the CNT-based sensor had a wide dynamic range of 10 nM^–1^ mM (Figure 4c). By fitting the curve, one can find the value of *K* as 1.4 × 10^6^ M^–1^. The result can be translated to *E*_0_~0.67 V, which was consistent with previously reported standard potential of swCNTs.

CNT-based sensors can be utilized for gas-sensing applications. For instance Kong et al. [51] developed a highly sensitive CNT-FET sensor for the detection of toxic gas molecules such as NO_2_ and NH_3_. A CNT-FET gas sensor which can detect formaldehyde gas was investigated by Kim et al. [53]. However, gas sensors based on bare CNTs have an innate limitation that such sensors often respond to the adsorption of non-specific molecules on to the CNTs, which limits its selectivity.

#### Biomolecule-immobilized CNT sensors

**Figure 5:**
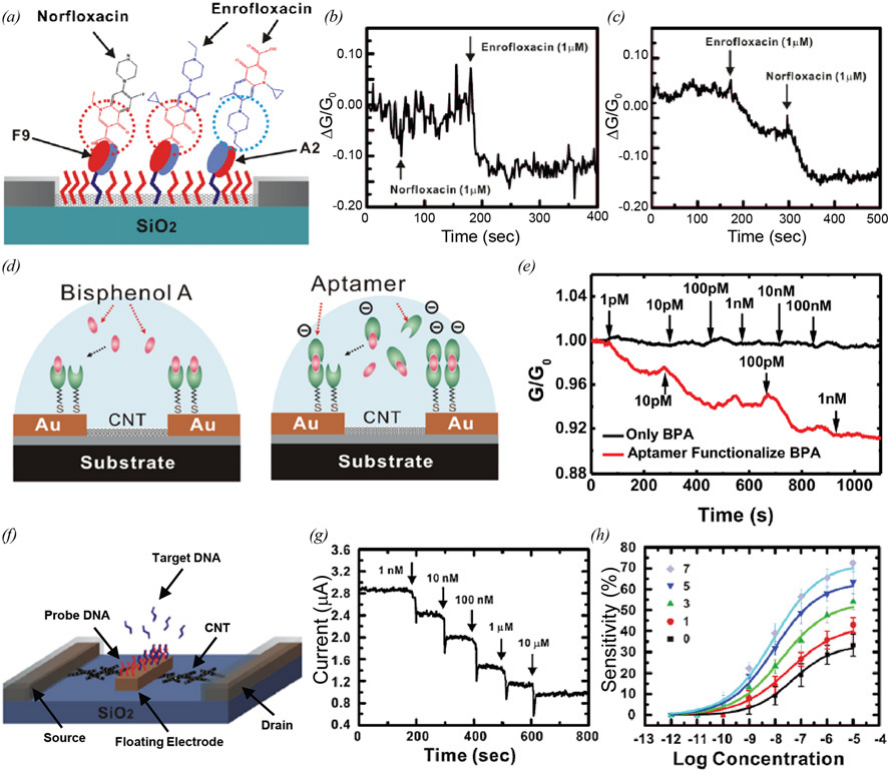
Bio-sensors comprised CNT-based hybrid nanostructures (**a**) Schematic diagram depicting the sensing experiment using antibody-immobilized CNT-FET sensors. Antibody fragment A2 specifically recognizes enrofloxacin antibiotics, while antibody fragment F9 recognizes fluoroquinolone family antibiotics including enrofloxacin and norfloxacin [54]. (**b**) Real-time conductance measurement using an A2-immobilized CNT-FET sensor. A2-immobilized CNT-FET sensors detected enrofloxacin only [54]. (**c**) Real-time conductance measurement using an F9-immobilized CNT-FET sensor. F9-immobilized CNT-FET sensors detected both enrofloxacin and norfloxacin [54]. (**d**) Schematic diagram showing the sensing experiment using aptamer-immobilized CNT-FET sensors. The left one is the sensing experiment of bare bisphenol-A (BPA), and the right one is the sensing experiment of aptamer-functionalized BPA [10]. (**e**) Real-time conductance measurements using aptamer-immobilized CNT-FET sensors. The aptamer-immobilized CNT-FET sensors showed recognizable response only to the injection of aptamer-functionalized BPA [10]. (**f**) Schematic diagram depicting the sensing experiment using DNA-immobilized CNT-FET sensors. The target DNA attached to the probe DNA on the surface of a floating electrode [56]. (**g**) Real-time current measurement using a DNA-immobilized CNT-FET sensor after the injection of target DNA at various concentrations [56]. (**h**) Response curves of DNA-immobilized CNT-FET sensors with various numbers of floating electrodes. The sensitivity increased with an increasing number of floating electrodes [56].

CNTs can be easily combined with various biological components, while maintaining the electrical properties of CNTs. CNT-FET devices coated with biomolecules have been utilized for the detection of biomolecular targets with a high selectivity. For example Kim et al. [54] reported a highly sensitive biosensor based on antibody fragment-immobilized CNTs for the family selective detection of antibiotics. The sensors are composed of CNT transistor transducers, linker molecules and antibody fragments as shown in Figure 5a. When target molecules are bound to antibody fragments, the negative charges of antibody fragments near the CNT surfaces are decreased. Since CNT-FETs exhibit p-type characteristics, the reduced negative charges near the CNT channel result in the decrease in a source-drain current in the channel. The authors used two different antibody fragments; A2 and F9. The A2 is the specific receptor of enrofloxacin which is one of antibiotics. The F9 is the general receptor of a fluoroquinolone family including enrofloxacin and norfloxacin. In A2-immobilized CNT sensors, 1 μM norfloxacin had no significant effect on the conductance of the CNT sensors, while a significant decrease in the conductance was detected after the injection of 1 μM enrofloxacin (Figure 5b). This indicates that the A2-immobilized CNT sensors selectively detect enrofloxacin in real time. On the other hand, F9-immobilized CNT sensors showed significant changes in the conductance after the injection of the 1 μM solutions of the enrofloxacin and norfloxacin (Figure 5c). The F9 recognized both enrofloxacin and norfloxacin that are the same antibiotics family. This result shows that F9-immobilized CNT sensors can be used for the *family selective* detection of the antibiotics. By choosing proper biomolecular receptors, versatile biosensor could be developed. Besteman et al. [13] developed glucose oxidase-immobilized CNT-FET sensors for the monitoring of glucose oxidation. The highly selective detection of streptavidin was demonstrated using biotin as a linker molecule [41]. In addition, highly selective CNT-FET biosensors using aptamers as recognition elements [47]. The performances of antibody- and aptamer-functionalized CNT-FET biosensors for the detection of target molecules have been compared [55].

One can functionalize the electrode parts in CNT-FETs with DNA molecules to build chemical or biological sensors with a high sensitivity and selectivity. Lee et al. [10] reported an aptamer sandwich-based CNT sensor for the selective and sensitive detection of BPA. This sensor contains aptamer molecules which are immobilized on the electrode parts of a CNT transistor (Figure 5d). When negatively charged target molecules bind to the aptamers on Au electrodes, the work function of the Au electrodes decreases, which increases the Schottky barrier height between the CNTs and the Au electrodes. The increase in the Schottky barrier height results in the change of the source-drain current. However, since BPA molecules are neutral, BPA molecules could not affect the conductance of the CNT-FET sensors (black line in Figure 5e). For the detection of neutral BPA, they functionalized BPA molecules with negatively charged DNA aptamers. In this case, after the injection of the functionalized BPA solutions (1–100 pM) to an aptamer-immobilized CNT-FET sensor, significant changes in the conductance of the CNT-FET sensor could be observed (red line in Figure 5e). Various other biosensors with DNAs on the electrode parts were also reported. Tang et al. [57] developed ssDNA-immobilized CNT-FET biosensors. The authors demonstrated direct label-free detection of DNA hybridization. CNT-FET sensors using DNA-immobilized electrodes were fabricated by Gui et al. [58]. The authors measured the effect of work function changes on the electrical detection of DNA hybridization.

#### CNT sensor structures with an improved performance

Many researchers have shown that the sensitivity of CNT-FET sensors can be enhanced by modifying the structure of the CNT-FETs. For example Kim et al. [56] reported a DNA sensor based on a *floating electrode* structure. The authors showed that the sensitivity of CNT-FET sensors could be enhanced simply by changing the number of floating electrodes. Figure 5f shows the schematic diagram depicting the structure of a floating electrode-based DNA sensor. Probe DNAs are immobilized on to the surface of the floating electrodes. When target DNAs were added, they bind to the probe DNAs on the floating electrodes and the negative charges in DNA backbones decrease the conductance of the CNT-FET through the Schottky barrier modulation mechanism. Figure 5g shows the response of a floating electrode-based sensor after the injection of target DNA solutions at various concentrations. The injection of target DNA molecules resulted in the decrease in the source-drain current in the floating electrode-based sensor. Figure 5h shows the sensitivity of the floating electrode-based sensors with respect to the number of floating electrodes, which clearly indicates that the sensitivities of the DNA sensors increase with the increasing number of floating electrodes. Similarly, increasing the Schottky contact areas of the device can improve the sensitivity of CNT-FET devices [59].

**Figure 6:**
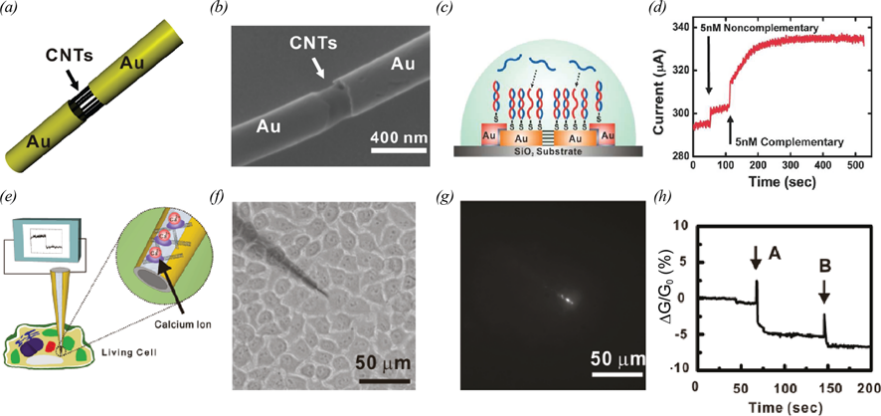
CNT sensor structures with an improved performance (**a**) Schematic diagram depicting the structure of an NBW [60]. (**b**) SEM image of an NBW [60]. (**c**) Schematic diagram showing the DNA sensing experiment using an NBW-based FET [60]. (**d**) Real-time current measurement using NBW-based FET after the injection of non-complementary DNA and complementary DNA molecules [60]. (**e**) Schematic diagram depicting the intracellular calcium ion sensing experiment using nanoneedle shape transistor-based sensor (NTS). Fluo-4-AM, a fluorescent calcium indicator, was immobilized on the CNT channel of an NTS for the detection of Ca^2+^ [15]. (**f**) Optical image of an NTS impenetrated into a HeLa cell [15]. (**g**) Fluorescence image of the NTS after adding 2 μM ionomycin into the extracellular medium [15]. (**h**) Real-time conductance measurement using an NTS after the injection of ionomycin [15].

Lee et al. [60] reported biosensors based on nanotube-bridged wires (NBWs) that are prepared by electrodeposition and on-wire-lithography (OWL) techniques. The *scheme* and the *SEM image* of an NBW are shown in Figure 6a and b respectively. In this study, NBWs were utilized as a part of DNA sensors (Figure 6c). Note that the DNA sensors negligibly responded to the injection of 5 nM non-complementary DNA sequences, while the injection of complementary DNA molecules resulted in the significant changes in the conductance of the DNA sensors at the same concentration of 5 nM (Figure 6d).

A nanoneedle shape transistor-based sensor (NTS) for the detection of intracellular calcium ions was developed by Son et al. [15]. In this work, a CNT-FET was fabricated at the end of a nanoneedle with a submicrometre diameter, and the CNT channel was functionalized with a Fluo-4-AM (Figure 6e). Fluo-4-AM is a fluorescence indicator for calcium ions. The binding of calcium ions to Fluo-4-AM leads to the emission of fluorescence lights, which gives the gating effects on the NTS. Figure 6f shows the bright-field image of HeLa cells impenetrated by the NTS. After the injection of 2 μM ionomycin, the NTS exhibited bright fluorescence at the end of the needle. This indicates the increase in the intracellular calcium concentration (Figure 6g)*.* The authors measured the source-drain current of the NTS to monitor the change of intracellular calcium concentration in a HeLa cell (Figure 6h). Here, the intracellular environmental changes by adding ionomycin lead to the increase in the intracellular calcium concentration.

### CNT-based artificial sensory system

#### Receptor-based sensory system

Signal transduction in a natural sensory system is triggered by the binding of odorant or tastant molecules to sensory receptors [61,62]. The sensory receptors can bind the targeted ordorant or tastant molecules with a high sensitivity and selectivity. Thus, the hybridization of sensory receptors and CNT-FETs gives us a new strategy for the fabrication of bioelectronic noses and tongues with a high sensitivity and selectivity.

**Figure 7:**
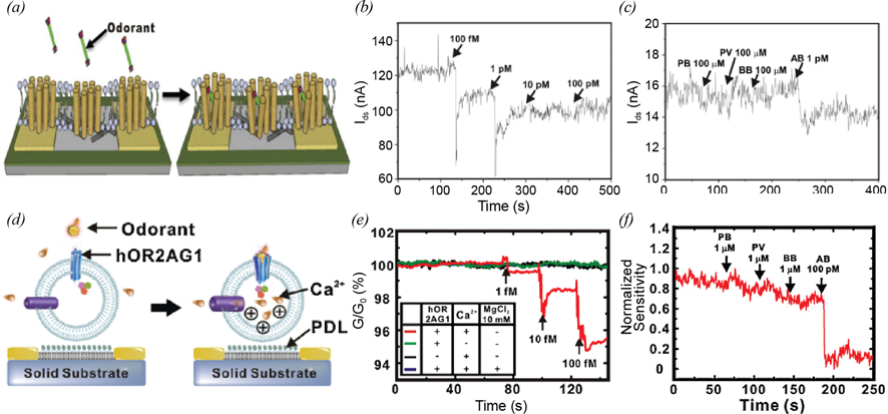
CNT-based bioelectronic nose and tongue (**a**) Schematic diagram depicting the plausible mechanism of olfactory receptor-immobilized CNT-FET sensors [17]. (**b**) Real-time current measurement using an olfactory receptor-immobilized CNT-FET sensor after the injection of AB at various concentrations [17]. (**c**) Real-time current measurement using an olfactory receptor-immobilized CNT-FET sensor after the injection of PB, PV, BB and AB [17]. (***d***) Schematic diagram depicting the plausible mechanism of olfactory nanovesicle-immobilized CNT-FET sensors. When a target odorant binds to hOR2AG1 protein, the signal pathways in the nanovesicle are activated. This induces the influx of Ca^2+^ through calcium ion channel. The increased positive charges in the nanovesicle give a field effect on an underlying CNT channel. Then, the conductance of the CNT channel decreases [18]. (**e**) Real-time conductance measurements using olfactory nanovesicle-immobilized CNT-FET sensors. The CNT-FET sensors showed response to the injection of 1 fM AB solution (red line). However, the two control experiments using nanovesicles without hOR2AG1 (black line) and PBS without Ca^2+^ (green line) showed no significant responses [18]. (**f**) Graph of the concentration-dependent responses of olfactory nanovesicle-immobilized CNT-FET sensors to AB solution [18].

Kim et al. [17] reported a bioelectronic nose based on a human olfactory receptor 2AG1 (hOR2AG1)-immobilized CNT-FET for the detection of odorant molecules with a single-carbon atomic resolution. The bioelectronic sensors are composed of CNT transistor transducers and immobilized olfactory receptors with a lipid membrane. Figure 7a shows the structure of a receptor-based sensor and the mechanism of the specific odorant detection using the CNT-FET sensor. When a specific odorant binds to its corresponding receptor, the state of the receptor may shift to the active state with negative charges. The negative charges in the receptor result in the increase in the energy level of CNT channel underneath the receptor [63,64]. Sequentially, the increase in the energy level of CNT channel may induce the increase in the Schottky barrier height of the Au-CNT junctions and the decrease in the CNT channel conductance. The hOR2AG1-immobilized CNT-FETs could detect amyl butyrate (AB), which is a kind of fruity odorants, down to 100 fM (Figure 7b). Non-targeted odorants, such as propyl butyrate (PB), pentyl valerate (PV) and butyl butyrate (BB), did not affect the conductance of a hOR2AG1-immobilized CNT-FET at 100 μM, whereas the addition of 1 pM AB resulted in a sharp decrease in the conductance (Figure 7c).

Using tastant receptors, a bioelectronic supertaster (BST) based on a human taste receptor-immobilized CNT-FET for the recognition of bitter tastants was investigated [65]. Goldsmith et al. [66] reported a method to integrate olfactory receptors with CNT-FETs for the development of olfactory gas sensors. Furthermore, chemically modified CNT-based biosensors with covalently attached olfactory receptors were reported [67]. Various kinds of receptors were utilized for the development of a multiplex bioelectronic device, mimicking the human sensory system [68]. These sensory receptor-based CNT-FETs have human-like sensitivity and selectivity.

#### Nanovesicle-based sensory system

In a cell, the binding of target molecules to sensory receptors increases the intracellular calcium concentration by the calcium signal pathway. According to the calcium signal pathway, cell membrane proteins, such as sensory receptors, adenylyl cyclases and ion channels are involved in the change of the intracellular calcium concentration. Since a nanovesicle derived from a cell partially contains the membrane proteins and its signal transduction pathways like a living cell, the nanovesicles can induce the influx of calcium ions by the calcium signal pathway when targeted molecules bind to the sensory receptors, which can be used to build selective sensors.

Jin et al. [18] developed a nanovesicle-based bioelectronic nose (NBN) platform by mimicking the signal pathways of human olfactory systems. The structure and mechanism of nanovesicle-based sensors are depicted in Figure 7d. NBNs are composed of CNT transistor transducers and immobilized nanovesicles containing olfactory receptors (hOR2AG1), adenylyl cyclases and ion channels. When odorant solutions are injected to nanovesicle-immobilized CNT-FETs, the binding events between olfactory receptors and odorant molecules trigger the calcium signal pathway. Then, the successive activation of olfactory receptors, adenylyl cyclases and ion channels induces the flux of calcium ions into the nanovesicles. The influx of calcium ions by the signal pathway gives a field effect on the underlying the CNT channel, thus resulting in the decrease in conductance of the CNT-FET. Based on this mechanism, the authors could detect AB, a specific odorant of hOR2AG1, down to 1 fM using the NBN platform (Figure 7e). The validity of the proposed mechanisms could be supported by the following two experiments. One experiment was conducted using nanovesicles not containing hOR2AG1. The other experiment was performed in calcium-free PBS. In the two experiments, there were no changes in the conductance of CNT-FETs. These indicate that both olfactory receptors and calcium ions are critical components for the operation of the NBNs. Figure 7f shows a real-time conductance measurement from an NBN device after the introduction of PB, PV, BB and AB. Conductance changes after injecting 1 µM of non-targeted odorants such as PB, PV and BB were negligible. After the injection of AB with 100 pM, the significant conductance change was observed. This result indicates that olfactory nanovesicle-immobilized CNT-FET sensors are highly selective and sensitive to target molecules.

Utilizing the platform based on NBNs, Park et al. [12] developed a bioelectronic sensor that can detect spoiled foods. The authors investigated the responses of the CNT-FET to hexanal that is an indicator of the oxidation of food. In this study, the bioelectronic sensor detected hexanal down to 1 fM with a high selectivity. In addition, this sensor allowed them to detect hexanal in spoiled milk. In general, spoiled milk contains a lot of hexanal resulting from the lipid oxidation. These results suggest the promising applications of canine olfactory receptor 5269 (cfOR5269)-immobilized bioelectronic sensors for the detection of decomposed foods. Lim et al. [69] reported the applications of a sensitive and selective NBN for the screening of lung cancer. Here, they detected heptanal, which is the odour of a lung cancer biomarker, down to 10 fM in the real-time measurement.

Song et al. [70] reported a bioelectronic tongue for the detection of sweetners using heterodimer taste receptors. This bioelectronic tongue could distinguish the nature and artificial sweetners just like human sensory systems. The sensor showed similar responses for the real samples with the standard sweetner solutions. A bioelectronic tongue for the discrimination of umami tastants was developed by Lee et al. [71]. The bioelectronic tongue was responded by the introduction of monosodium glutamate (MSG) down to 100 pM. Moreover, this device showed the synergistic effect by the disodium 5′-inoinate (IMP) to enhance the detection of MSG.

## Cell control

The controls of cellular behaviours, such as adhesion, proliferation and differentiation, are important issues in cell therapies and tissue engineering. Recently, synthetic bio-inspired materials have been applied to the controls of cell shapes and growth behaviours. In particular, CNT-based hybrid nanostructures exhibit their promising results in cell controls. In this chapter, we will review the applications of CNT-based hybrid nanostructures for the control of cell shape, growth and differentiation. In addition, cytotoxicity and biocompatibility of CNT-based devices will be discussed.

### Control of cell shapes using CNT patterns and alignments

Cell shapes can be determined by various physical forces including resting force (residual strain) and additive mechanical forces (deformational loading) [22]. Note that the cell shape is one of important factors for cell development and physiology [22,72]. Thus, the control of cell shapes via various cues such as physical cues, growth factor and extracellular matrix (ECM) proteins can be a means to alter the growth, differentiation, apoptosis and migration of cells.

**Figure 8:**
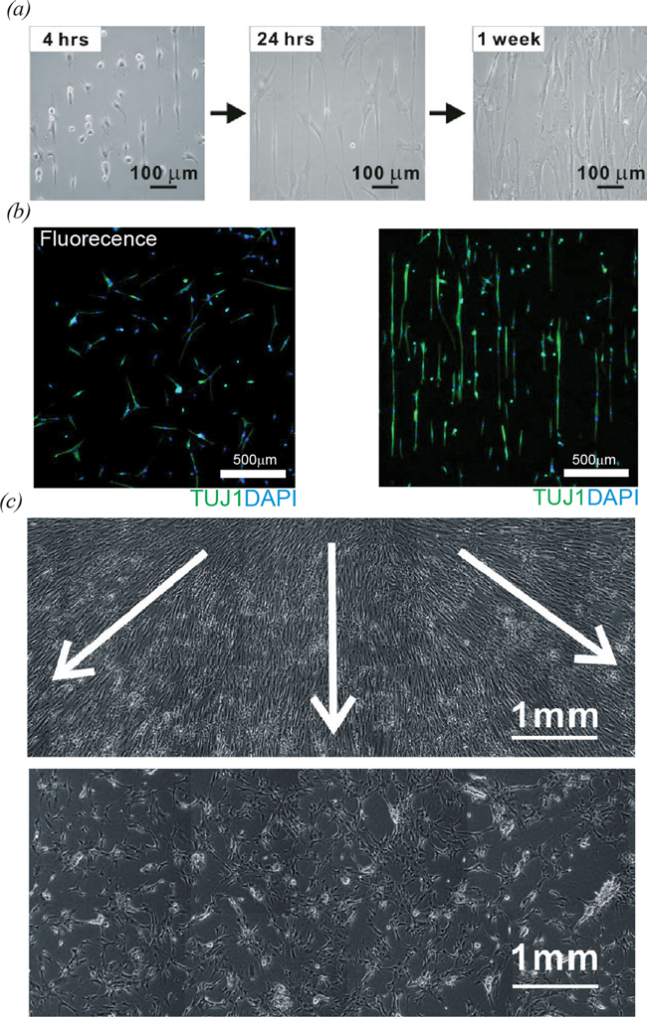
Cell growth behaviours on CNTs (**a**) The bright field images of MSCs on CNT patterns [8]. (**b**) Fluorescence images of the hMSCs differentiated after 2 weeks of culture on bulk CNT network films (left) and linear CNT network patterns (right). Immunostaining markers were TUJ1 (green) for neural cells and DAPI (blue) for nuclei [26]. (**c**) Phase contrast image of hMSCs cultured on the aligned CNT networks for 2 weeks. The white arrows indicate the radial direction of the CNT spin coating. The images showed directional growth of hMSCs following CNT alignments [73].

Recently, several researchers have been investigated to control the shapes of stem cells using CNT patterns and alignments. Park et al. [8] reported a method for the controls of mesenchymal stem cell (MSC) shapes and growth behaviour using CNT monolayer patterns (Figure 8). The CNT-patterned substrates consist of 1-octadecanethiol (ODT)-coated spacing regions (50 µm) and linear CNT patterns of 20 µm in width that is comparable with the sizes of individual MSC cell bodies. Figure 8a shows MSCs cultured on linear CNT patterns. Bright-field images clearly show that MSCs are preferentially adsorbed on the CNT region and are elongated along the linear CNT patterns with increasing culturing time. Cell shapes analysis confirmed that MSCs on the linear CNT-patterns were significantly elongated as compared with MSCs on non-patterned bulk CNT networks. The directed growth of MSCs along CNTs was explained by the strong affinity of CNTs to cell adhesion proteins, such as fibronectin (FN). Interestingly, linear CNTs patterns could enable the elongation of stem cell nuclei along the patterns. For example hMSCs on linear CNT pattern exhibited low circularity of their nuclei as compared with hMSCs on bare glass and bulk CNT networks. On the other hands, linear CNT patterns can control the differentiation of stem cells. hMSCs on linear CNT patterns exhibited elongated cell shapes and maintained their shapes along the CNT line patterns after differentiation (Figure 8b). Here, the neural gene expressions of hMSCs on the linear CNT patterns were measured significantly higher than that on the bulk CNT networks. The authors proposed a plausible explanation that the elongations of the hMSCs on the linear CNT patterns resulted in the enhanced MAP2 expression compared with those on the bulk CNT networks. The directional growth of other cells along CNT pattern was also reported. For example rat hippocampal neurons on poly-L-lysine-coated CNT patterns exhibited the directional growth of neurites along the CNT patterns. In addition, hMSCs cultivated on linear CNT patterns functionalized with FN presented the selective filopodial extension along the CNT patterns. On CNT line patterns functionalized laminin, human neural stem cells (hNSCs) were found to align to form a bipolar shape during the growth and differentiation.

Alignments of CNTs can not only control the shapes of stem cells but also affect the proliferation and differentiation of stem cells. Like linear-shaped CNT patterns, aligned individual CNTs were found to induce the elongation and directional growth of stem cells along CNTs. Namgung et al. [73] reported a method to control the growth behaviour of hMSCs using the alignment of individual CNTs. Figure 8c shows morphology of cells cultured on the aligned or randomly oriented CNTs. The cells on the aligned CNTs grew in radial direction along the individual CNTs, while the hMSCs on randomly oriented CNTs were aggregated and spread randomly. Interestingly, hMSCs recognized individual CNTs even with its sub-2-nm diameter, and they directionally grew along the aligned CNTs. The elongated and stretched shape of hMSCs was explained by the alignment of actin filaments along the CNTs. In addition, hMSCs on aligned CNTs exhibited a significantly enhanced proliferation compared with those on randomly oriented CNTs. From quantitative real-time PCR (qPCR) analysis, the authors found that hMSCs on the aligned CNTs significantly up-regulated the levels of osteogenic gene expressions, such as osteopontin, osteocalcin (OCN) and alkaline phosphate, compared with those on the randomly oriented CNTs. As a plausible explanation for the up-regulated osteogenic gene expressions of the arranged hMSCs, the authors proposed mechanotransduction pathways triggered by high cytoskeletal tension exerted on the elongated and arranged hMSCs. Similarly, Galvan-Garcia et al. [74] observed the directed cell growth along highly oriented CNT sheets and yarns. In this work, the migration of fibroblasts on CNT sheets was found to be enhanced.

### Controls of differentiation and other cell growth behaviour

Due to mechanically strong and chemically stable properties of CNTs, CNT-based hybrid nanostructures have been utilized to control versatile cellular responses including enhanced growth and differentiation of cells on CNT surfaces.

**Figure 9:**
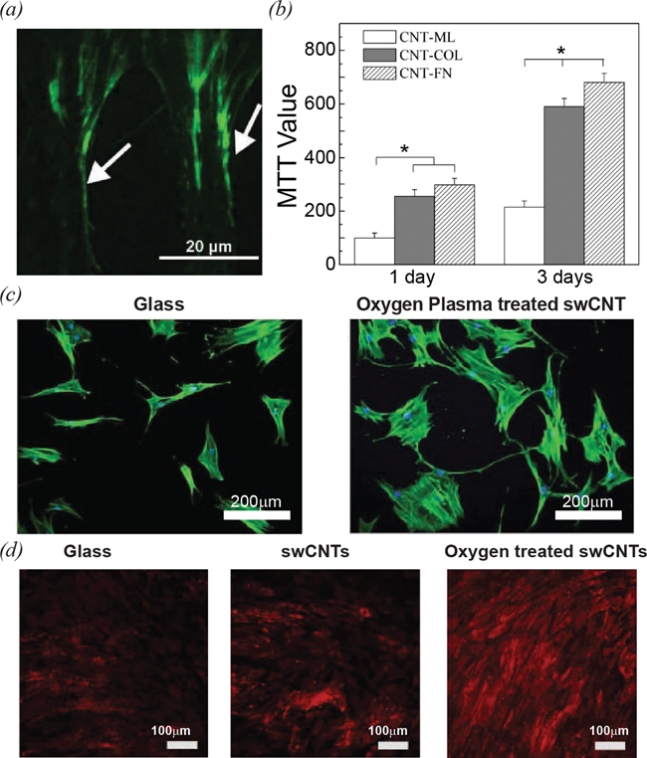
Cell growth on CNT-based hybrid nanostructures (**a**) Total internal reflection fluorescence (TIRF) image of immunostained vinculins (green) of hMSC. The white arrows indicate the focal adhesion sites where the selective filopodial extensions are observed [22]. (**b**) The histogram of MTT test result for PEFs on CNT monolayer (CNT-ML), collagen-functionalized CNT (CNT-COL), fibronectin-functionalized CNT (CNT-FN). These indicate significantly enhanced viability of PEFs on CNT-COL and CNT-FN [75] (**c**) Fluorescence images of actin filaments (green) of hMSCs on a glass substrate (left) and an oxygen plasma-treated swCNT (O-swCNT) monolayer (right) [21]. (**d**) Immunohistochemistry of an osteogenic protein of hMSCs on glass substrates, swCNT substrates and O-swCNT substrates at day 12 [21].

CNTs coated with ECM proteins can promote cell adhesion and induce cytoskeleton remodelling and morphological changes of cells. Namgung et al. [22] investigated the adhesion properties and conformational change of FN on CNTs via immunofluorescence microscopy and force-spectroscopy analyses. In this work, FNs were coated on CNT networks to prepare FN-CNT hybrid nanostructures. CNT functionalized with FN exhibited a stronger affinity to hMSCs or HeLa cells than bare CNTs and FN-adsorbed glass substrates, resulting in highly selective filopodial extensions along the FN-adsorbed CNTs (Figure 9a). It was found that CNT monolayers immobilized with collagen and FNs increased spreading area, adhesion energy and proliferation rate of primary porcine oesophageal fibroblast (PEF), which in turn led to promotion of cell adhesion and proliferation (Figure 9b) [75]. Interestingly, PEFs cultured on collagen or FN-immobilized CNTs exhibited enhanced elongation compared with PEFs on bare CNTs. To date, it is not fully understood why CNTs functionalized with ECM proteins improved the adhesion properties of cells, but strong and effective adhesion of cells on to CNT-based devices can be advantageous for the applications of tissue regeneration.

The cell growth and differentiation can be controlled by plasma-treated CNT patterns [76,77]. Baik et al. [21] investigated the effect of oxygen plasma-treated CNT patterns on the growth behaviours of hMSCs. The hMSCs cultured on the oxygen plasma-treated CNT substrates showed enhanced cell adhesion areas and proliferation compared with those on glass and pristine CNTs (Figure 9c). The improved proliferation and adhesion of hMSCs was explained by the changes in surface chemistry of the plasma-treated CNT substrates such as enhanced hydrophilicity and surface oxygen content [76]. Furthermore, oxygen plasma treatment on CNTs was found to enhance the osteogenic differentiations of hMSCs. hMSCs on the oxygen plasma-treated swCNTs exhibit the brighter fluorescence of OCN that is one of osteogenic proteins (Figure 9d). The improved alkaline phosphatase (ALP) activity of hMSCs on the plasma-treated swCNTs supports the enhanced osteogenic differentiations of hMSCs. The enhanced osteogenesis of hMSCs on the oxygen treated CNT surfaces was attributed to the stress on cells due to enhanced cell spreading. However, extensive oxygen plasma treatments were found to have negative effects on cell growth on a CNT layer. Kalbacova et al. [77] found that the 5 min of oxygen plasma treatment significantly enhanced the growth and adhesion behaviours of osteoblasts on a swCNT layer, while 30 min of the treatment reduced the osteoblast adhesion. The reduced cell adhesion was explained by the surface morphology changes of the CNT layer after excessive treatment of oxygen plasma.

On the other hand, CNT-based hybrid devices can be used to transmit electrical stimulation to control the growth of neural cells [24,78,79]. Huang et al. [25] investigated the effect of electrical stimulations on the growth and differentiation of neural stem cells (NSCs) on CNT ropes. The low electrical stimulation (<10 mV) was found to have negligible effect on the viability of NSCs, but promote the extension of neurite outgrowth. In addition, the electrical stimulation was found to have a boosting effect on the differentiations of NSCs into more mature neurons. The effect of electric fields on the adhesion of human neuronal cells (neuroblastoma SHSY5Y) and 3D patterns of swCNT was investigated by Dionigi et al. [24]. The 3D patterns of swCNT with an electric field above 1 V cm^−1^ were found to enhance the adhesion of the cells. However, the enhanced cell adhesion was depleted at 5 V/cm. Wang et al. [79] developed a substrate for neural interfaces using vertically aligned mwCNT pillars. The neurons were grown and differentiated on the hydrophilic functionalized CNT microelectrodes, and they were repeatedly excited with charge-unbalanced stimulation protocols. Interestingly, the CNT electrodes operated predominantly with capacitive currents without faradic reactions, which were considered ideal for a neural stimulation. Different from conventional devices for electrical stimulations, CNT-based electrodes allow us to localize electric fields on desired healing area [80]. The local electrical stimulation via CNT-based electrodes has a great benefit in clinical application by reducing the unexpected exposure of electrical stimulation to the body.

### Cellular responses to CNT composite solutions

CNTs can penetrate into living cells when the cells are cultured in aqueous suspensions of CNTs [81,82]. Furthermore, CNTs functionalized with biomolecules can penetrate more easily into cells than pristine CNTs [83]. Kam et al. [84] reported a method for the cellular uptake of proteins and DNAs using CNTs. In this work, HeLa and HL60 (human promyelocytic leukemia) cells were cultured in growth media containing CNTs that were non-covalently conjugated with proteins and DNAs. The proteins and DNAs were labelled with fluorophores for the observation of cellular uptakes. CNTs successfully transported DNAs and proteins into living cells via confocal microscopy images and flow cell cytometry measurements. Various biomolecules can be transported into living cells via a similar strategy. A transportation method of RNA into breast cancer cells (MCF7) using swCNTs has been studied by Lu et al. [85]. In addition, Pantarotto et al. [86] succeeded in the transport of bioactive peptide into human fibroblast using CNTs. The transport of DNAs and RNAs provide great chances for gene therapies and disease preventions although the cytotoxicity of CNTs is still left to be solved prior to practical applications.

The toxicity of CNTs has been extensively studied [81–83]. The uptake of CNTs in cells was found to induce the disruption of intracellular metabolic pathways, oxidative stresses or physical membrane damages causing cell ruptures. Magrez et al. [82] demonstrated that CNTs suspended in a medium with gelatin inhibited cell proliferation. In this study, the toxicity of CNTs increased when they were functionalized with carbonyl, carboxy and hydroxyl groups. In addition, the physical properties of CNTs, such as length and rigidity, may play important roles in the cytotoxicity of CNTs. Indeed, several experimental evidences indicated that long (>20 μm) and rigid CNTs should be not used for *in vivo* and clinical applications [81–83].

## Summary

Recently, CNT-based hybrid devices have been intensively studied for biological interfaces. Versatile nanoscale hybrid structures based on CNTs can be fabricated by various methods including surface-programmed assembly and gas glow controlled growth. The nanoscale CNT patterns can be functionalized by chemical or biological molecular species to build highly sensitive and selective sensors. Furthermore, CNT patterns can be utilized to control the shape and growth of cells and its signal pathways. Overall, the unique electrical and mechanical properties of CNTs allow one to envision great opportunities for various applications such as quick diagnostics, artificial sensory systems and tissue engineering. However, for such practical applications, it should be critical to address various issues such as reliability of CNT-based biosensors and toxicity of CNTs for tissue engineering.

## Abbreviations

AB: amyl butyrate
BB: butyl butyrate
BPA: bisphenol-A
cfOR5269: canine olfactory receptor 5269
CNT: carbon nanotube
CNT-COL: collagen-functionalized carbon nanotube
CNT-FN: fibronectin-functionalized carbon nanotube
CNT-ML: carbon nanotube monolayer
CVD: chemical vapour deposition
ECM: extracellular matrix
FET: field-effect transistor
FN: fibronectin
hMSC: human mesenchymal stem cell
HL60: human promyelocytic leukemia cell
hOR2AG1: human olfactory receptor 2AG1
MSC: mesenchymal stem cell
MSG: monosodium glutamate
mwCNT: multi-walled carbon nanotube
NBN: nanovesicle-based bioelectronic nose
NBW: nanotube-bridged wire
NSC: neural stem cell
NTS: nanoneedle shape transistor-based sensor
OCN: osteocalcin
O-swCNT: oxygen plasma-treated single-walled carbon nanotube
OTS: octadecyltrichlorosilane
PB: propyl butyrate
PEF: porcine oesophageal fibroblast
PSE: 1-pyrenebutanoic acid succinimidyl ester
PV: pentyl valerate
SAM: self-assembled monolayer
swCNT: single-walled carbon nanotube
TIRF: total internal reflection fluorescence
π-stacking: aromatic-aromatic interaction
